# Supplementary material for: The “Bagno dell’Acqua” Lake as a Novel Mars-like Analogue: Prebiotic Syntheses of PNA and RNA Building Blocks and Oligomers
Source: Int J Mol Sci. 2025 Jul 19;26(14):6952. doi: 10.3390/ijms26146952 (PMC12295259; doi:10.3390/ijms26146952)
Supplement: Supplementary file 1 [file ijms-26-06952-s001.zip › ijms-3564134-supplementary.pdf]

The "Bagno dell'Acqua" Lake as a novel Mars-like analogue: prebiotic syntheses of PNA and RNA building blocks and oligomers

Valentina Ubertini[1], Eleonora Mancin[1], Enrico Bruschini[2], Marco Ferrari[2], Agnese Piacentini[3, 4], Stefano Fazi[3,4,], Cristina Mazzoni[3, 4] Bruno Mattia Bizzarri[1]*, Raffaele Saladino[1] and Giovanna Costanzo [2,5]*

[1] Department of Biological and Ecological Sciences (DEB), University of Tuscia, 01100 Viterbo, Italy; valentina.ubertini@unitus.it (V. U.); eleonora.mancin@unitus.it (E. M.); bm.bizzarri@unitus.it (B.M.B.); saladino@unitus.it (R. S.)
[2] Institute for Space Astrophysics and Planetology – INAF, 00133 Rome, Italy enrico.bruschini@inaf.it (E.B.); marco.ferrari@inaf.it (M.F.)
[3] Department of Biology and Biotechnology "C. Darwin", Sapienza University of Rome, 00185 Rome, Italy; agnese.piacentini@uniroma1.it (A.P.); cristina.mazzoni@uniroma1.it (C.M.)
[4] Water Research Institute, National Research Council (IRSA-CNR), Montelibretti, 00010 Rome, Italy; stefano.fazi@irsa.cnr.it (S.F.)
[5] Institute of Molecular Biology and Pathology, National Research Council - IBPM-CNR, 00185 Rome, Italy; giovannamaria.costanzo@cnr.it (G.C.)
* Correspondence: giovannamaria.costanzo@cnr.it; Tel.: +39 06 49912897; bm.bizzarri@unitus.it

SI #1: Characterization of the microbialite sample

Microbialite from the northeastern sector of the lake (C1) has been collected along the shoreline and it contains a large variety of mineral assemblage: aragonite (65% wt), quartz (2% wt), smectite (3% wt), plagioplase (8% wt), K-felspar (6% wt) and halite (15% wt) The bulk mineralogical composition of microbialite was determined by X-ray diffraction (XRD) analysis using a Bruker D8 Advance X-ray system equipped with a Lynxeye XE-T silicon-strip detector at the Department of Earth Sciences, Sapienza University of Rome [50]. Sample was dried in an oven at a temperature of 40 ◦C overnight. Then, 2 g of microbialite was gently crushed in agate mortar and random-ordered specimens were prepared and run between 2 and 70° 2θ with step sizes of 0.02° 2θ, 1 s per step (1 h and 6 min long scan), while spinning the sample at 40 kV and 30 mA using CuKα radiation (λ = 1.5406 Å). Data were collected with variable slit mode to keep the irradiated area on the sample surface constant and converted to fixed slit mode for semiquantitative analysis. Identification and semiquantitative estimation of mineral phases were performed using the software Diffrac.EVA 5.2 by calculating peak areas and using mineral intensity factors as calibration constants.

SI #2: Ions abundance and MS fragmentation profiles of products (4-27)

Table S1: m/z values, peak abundances, fragmentation profiles of compounds 4-27 and comparison of retention time with related standards.

Product	RT of selected reaction product[(a)]	RT of standard compound	m/z
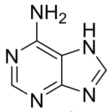 **(4)**[(f)]	**12.87**[(d)]	**12.85**	207 [M+ TMS] (42), 192[M+ TMS-Me] (100)
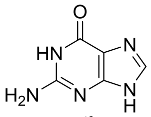 **(5)**[(f)]	**21.04**[(d)]	**21.12**	295 [M+ 2TMS] (38), 250[M+ 2TMS-3Me] (100), 223 [M+TMS]
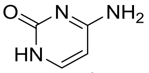 **(6)**[(f)]	**11.20**[(d)]	**11.15**	255 [M+ 2TMS] (25), 240[M+2TMS-Me] (30)
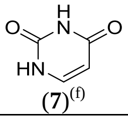 **(7)**[(f)]	**9.82** [(c)]	**9.80**	256 [M+ 2TMS] (58), 241 [M+ 2TMS-Me] (100), 147 [$(CH_3)_3$-Si-O=$Si(CH_3)_2^+$] (25), 112 [M] (12)
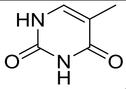 Thymine **(8)**[(f)]	**7.50**[(b)]	**7.53**	270 [M+ 2TMS] (30), 255 [M+ 2TMS-Me] (100), 147 [$(CH_3)_3$-Si-O=$Si(CH_3)_2^+$] (25), 73 [$(CH_3)_3$-Si^+)] (20)
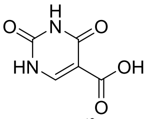 **(9)**[(f)]	**10.46**[(d)]	**10.40**	228 [M+TMS](30), 213 [M+TMS-Me](50), 156 [M] (15), 147 [$(CH_3)_3$-Si-O=$Si(CH_3)_2^+$] (35), 73 [$(CH_3)_3$-Si^+)] (100)
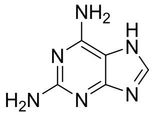 **(10)**[(f)]	**4.65**[(d)]	**4.70**	294 [M+2TMS] (40), 279[M+2TMS-Me] (100), 222 [M+TMS] (15), 207 [M+TMS-Me] (40)
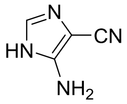 **(11)**[(g)]	**6.81**[(d)]	**6.81**	25 [M+2TMS+H] (5), 147 [$(CH_3)_3$-Si-O=$Si(CH_3)_2^+$] (40), 73 [$(CH_3)_3$-Si^+)] (100)
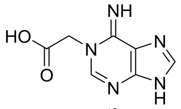 **(12)**[(f)]	**16.89**[(c)]	**16.90**	266 [M+1TMS] (5)
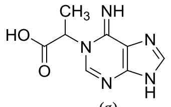 **(13)**[(g)]	**11.06**	**11.05**	278 [M+1TMS -H] (8), 207 [M] (2), 73 [$(CH_3)_3$-Si^+)] (100)
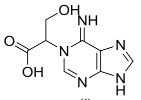 **(14)**[(i)]	**13.49**	**13.52**	443 [M+3TMS+3H] (25), 369 [M+2TMS+2H] (2), 353 [M+2TMS-Me] (20), 147 [$(CH_3)_3$-Si-O=$Si(CH_3)_2^+$] (10), 73 [$(CH_3)_3$-Si^+)] (100)

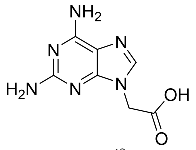 **(15)**(f)	**10.11**(d)	**10.09**	356 [M+2TMS] (5), 282 [M+1TMS] (5), 252 [M+1TMS-2Me] (20), 238 [M+1TMS-3Me] (8), 208 [M-H] (3), 73 [$(CH_3)_3$-Si^{+})] (100)
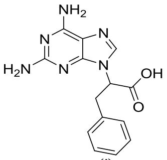 **(16)**(j)	**9.79**	**9.82**	299 [M+H] (20), 298 [M] (100)
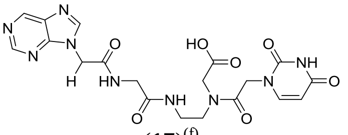 **(17)**(f)	**20.28**	**-**	487 [M+H] (100), 429 [M-Gly+H] (95), 147 [$(CH_3)_3$-Si-O=$Si(CH_3)_2^{+}$] (45), 73 [$(CH_3)_3$-Si^{+})] (100)
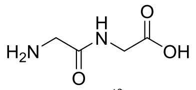 **(18)**(f)	**6.99**(e)	**7.00**	424 [M+ 4TMS] (2), 350 [M+ 3TMS] (2), 277 [M+2TMS] (95), 204 [M+1TMS] (2), 174 [M+1TMS-Me] (85), 147 [$(CH_3)_3$-Si-O=$Si(CH_3)_2^{+}$] (20), 132 [M] (8), 73 [$(CH_3)_3$-Si^{+})] (100)
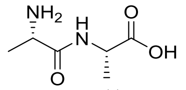 **(19)**(g)	**4.34** (d)	**4.36**	381 [M+ 3TMS+2H] (100), 307 [M+2TMS+H] (50), 291 [M+2TMS-Me] (20), 233 [M+1TMS] (15), 188 [M+1TMS-3Me] (45), 73 [$(CH_3)_3$-Si^{+})] (95)
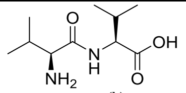 **(20)**(h)	**8.61**(c)	**8.65**	360 [M+2TMS] (2), 244 [M+1TMS-3Me] (30), 216 [M] (10), 73 [$(CH_3)_3$-Si^{+})] (100)
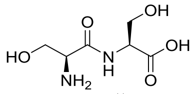 **(21)**(i)	**10.98**(c)	**10.91**	336 [M+2TMS] (55), 249 [M+1TMS-Me] (100), 192 [M] (8), 147 [$(CH_3)_3$-Si-O=$Si(CH_3)_2^{+}$] (45), 73 [$(CH_3)_3$-Si^{+})] (55)
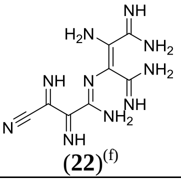 **(22)**(f)	**7.27**(b)	**-**	321 [M+1TMS] (50), 293 [M+1TMS-CN] (100), 147 [$(CH_3)_3$-Si-O=$Si(CH_3)_2^{+}$] (40), 73 [$(CH_3)_3$-Si^{+})] (40)
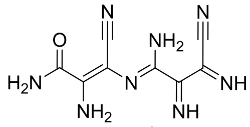 **(23)**(f)	**8.55**(b)	**-**	304 [M+1TMS] (2), 232 [M+2H] (100), 147 [$(CH_3)_3$-Si-O=$Si(CH_3)_2^{+}$] (40), 73 [$(CH_3)_3$-Si^{+})] (40)
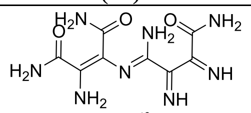 **(24)**(f)	**13.30**(c)	**-**	342 [M+1TMS] (100), 326 [M+1TMS-Me-H] (15), 73 [$(CH_3)_3$-Si^{+})] (35)
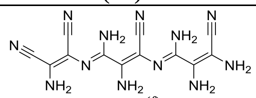 **(25)**(f)	**12.08**(c)	**-**	398 [M+1TMS] (30), 325 [M] (15), 73 [$(CH_3)_3$-Si^{+})] (80)
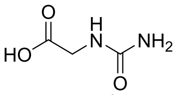 **(26)**(f)	**6.20**(b)	**6.25**	190 [M+1TMS] (100), 174 [M+1TMS-Me-H] (15), 147 [$(CH_3)_3$-Si-O=$Si(CH_3)_2^{+}$] (85), 73 [$(CH_3)_3$-Si^{+})] (15)
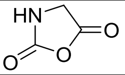	**8.33**(c)	**8.31**	245 [M+ 2TMS] (85), 147 [$(CH_3)_3$-Si-O=$Si(CH_3)_2^{+}$] (25),

(**27**)[(f)]			101 [M] (25), 73 $[(CH_3)_3$-$Si^+)]$ (55)

The abundance of ions is shown in round brackets. [a] Retention time (RT) expressed in minutes. Products have been detected with a different degree of silylation: [b] mono-silyl derivative; [c] di-silyl derivative; [d] tri-silyl derivative; [e] tetra-silyl derivative. [f] Fragmentation spectrum deriving from reaction A. [g] Fragmentation spectrum deriving from reaction B. [h] Fragmentation spectrum deriving from reaction C. [i] Fragmentation spectrum deriving from reaction D. [j] Fragmentation spectrum deriving from reaction E.

SI #3: Experimental m/z fragmentation spectra of products (4-27)

Original fragmentation spectra of compound 4 deriving from reaction A

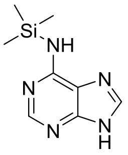

(**4**)

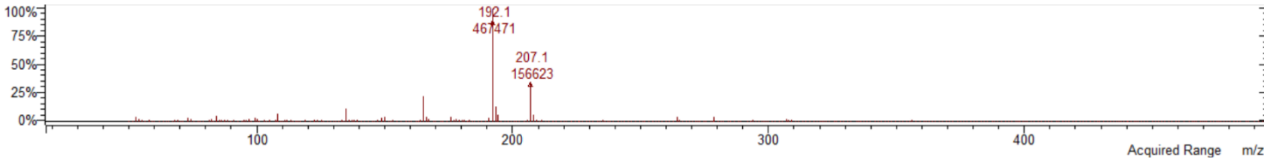

Original fragmentation spectra of compound 5 deriving from reaction A

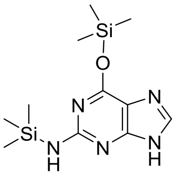

(**5**)

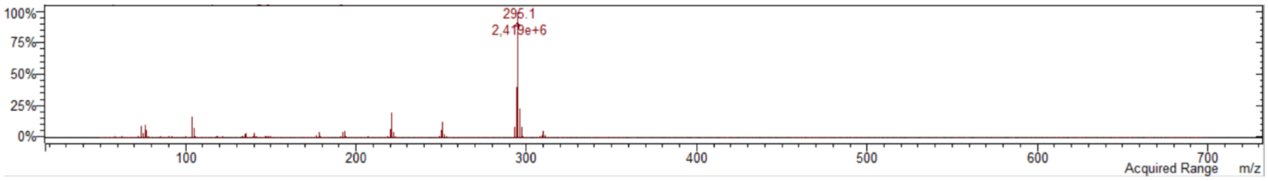

Original fragmentation spectra of compound 6 deriving from reaction A

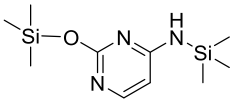

(**6**)

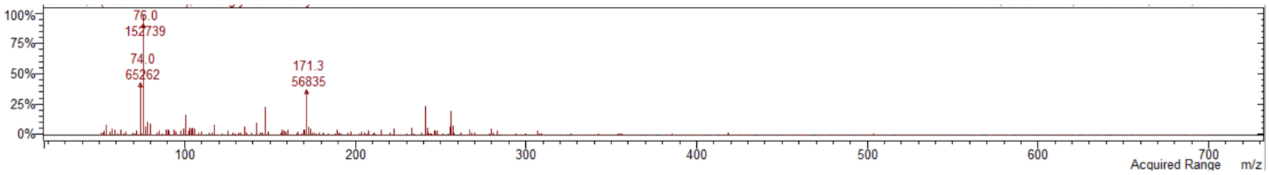

Original fragmentation spectra of compound 7 deriving from reaction A

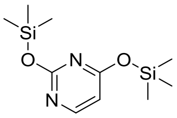

(**7**)

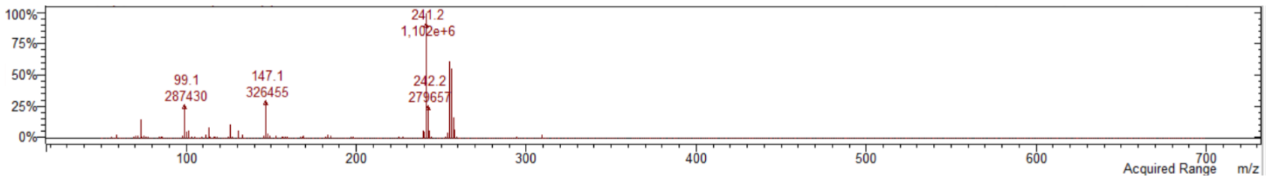

Original fragmentation spectra of compound 8 deriving from reaction A

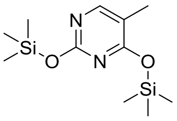

(**8**)

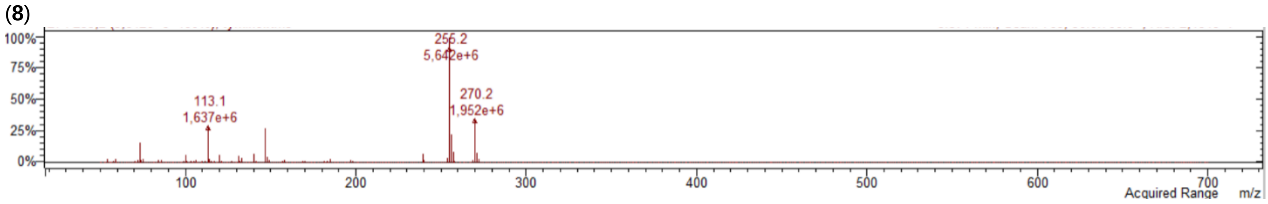

Original fragmentation spectra of compound 9 deriving from reaction A

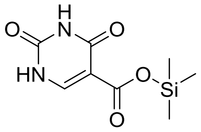

(**9**)

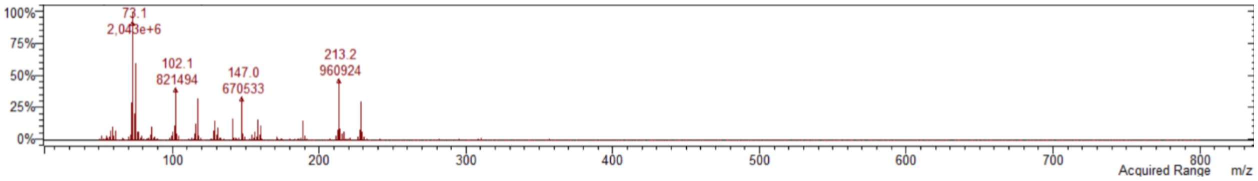

Original fragmentation spectra of compound 10 deriving from reaction A

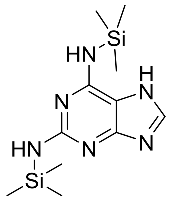

(**10**)

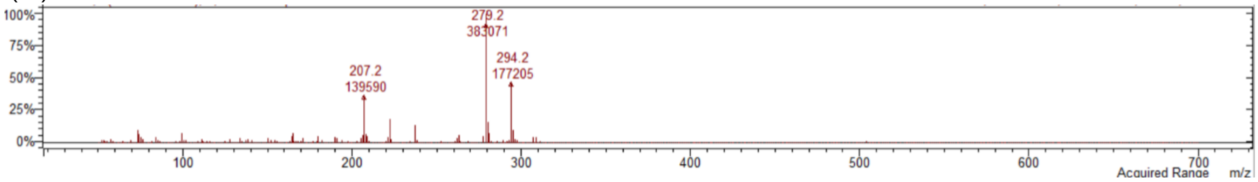

Original fragmentation spectra of compound 11 deriving from reaction B

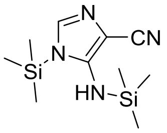

(**11**)

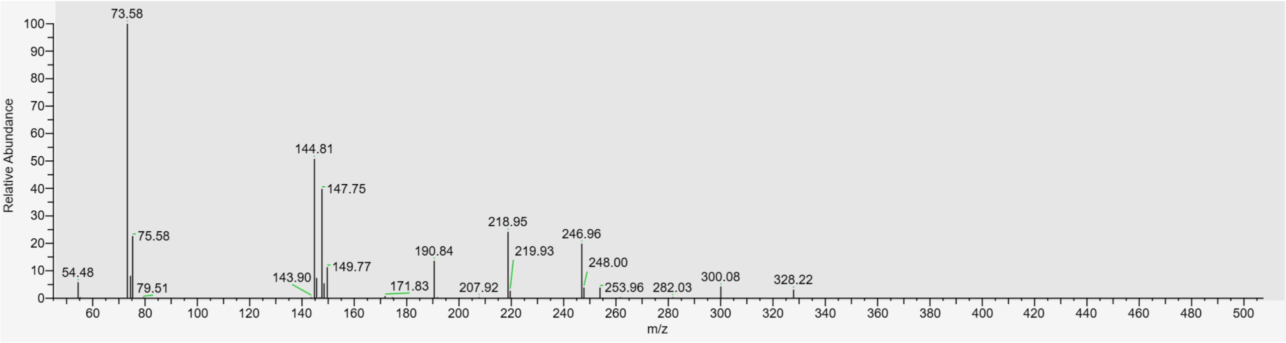

Original fragmentation spectra of compound 12 deriving from reaction A

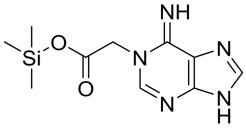

(**12**)

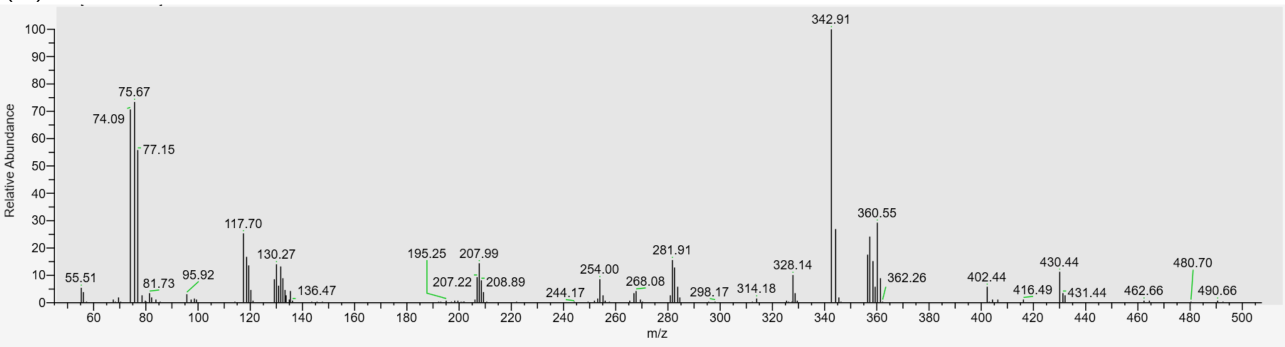

Original fragmentation spectra of compound 13 deriving from reaction B

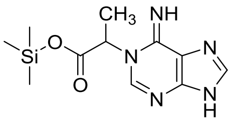

(**13**)

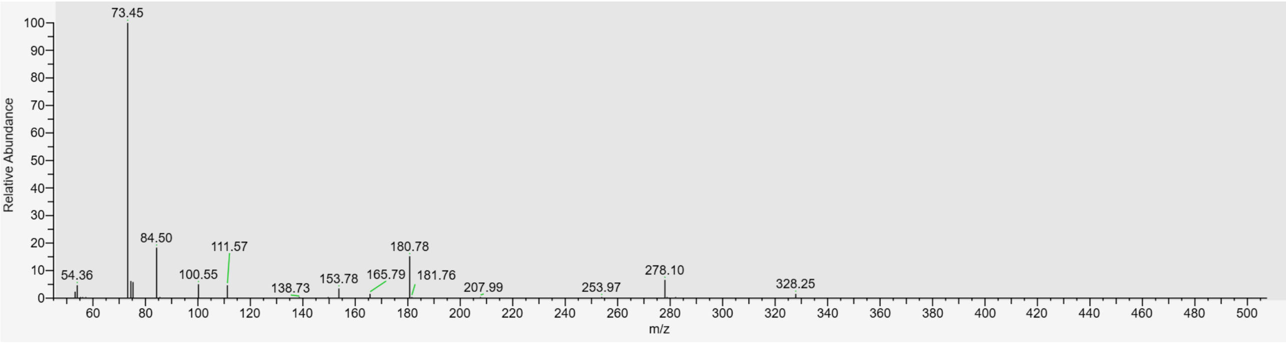

Original fragmentation spectra of compound 14 deriving from reaction D

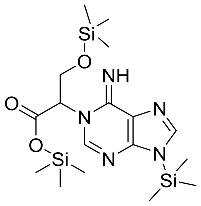

(**14**)

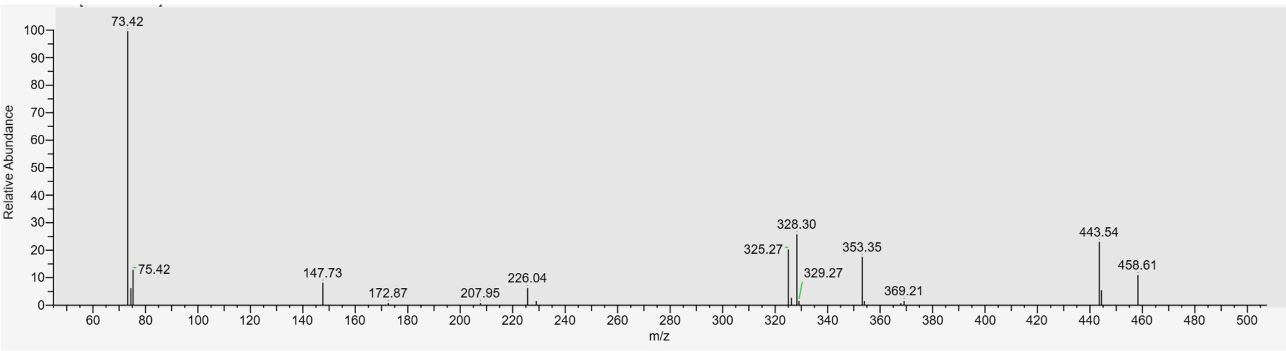

Original fragmentation spectra of compound 15 deriving from reaction A

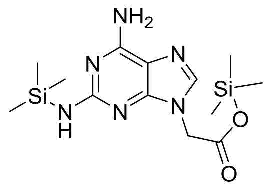

(**15**)

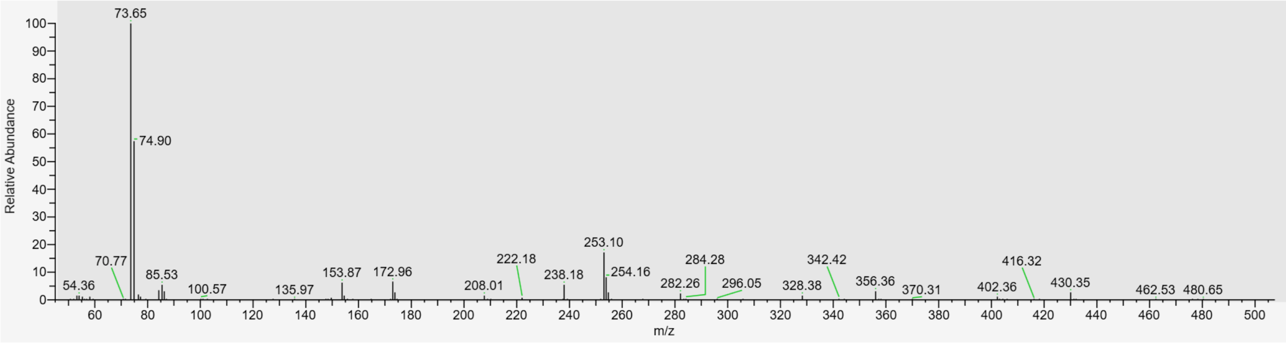

Original fragmentation spectra of compound 16 deriving from reaction E

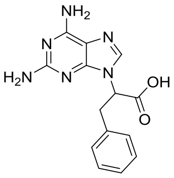

(**16**)

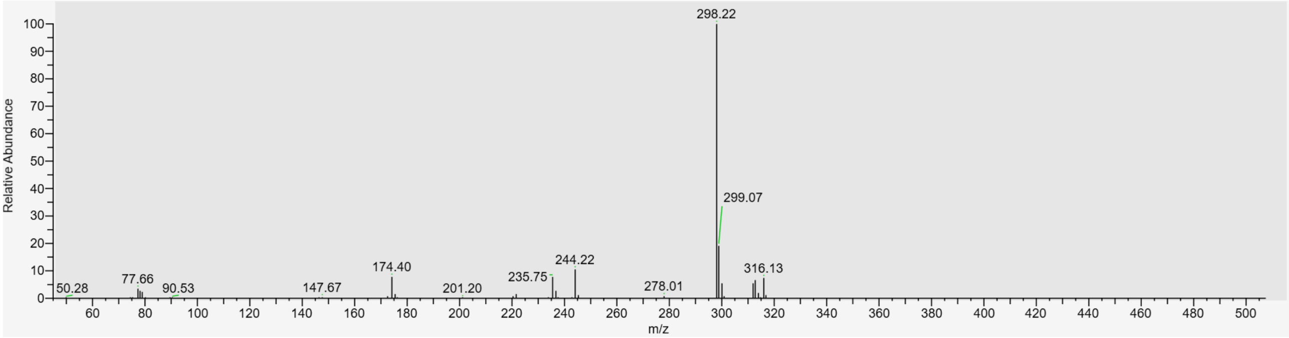

Original fragmentation spectra of compound 17 deriving from reaction A

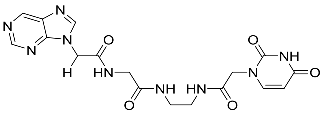

(**17**)

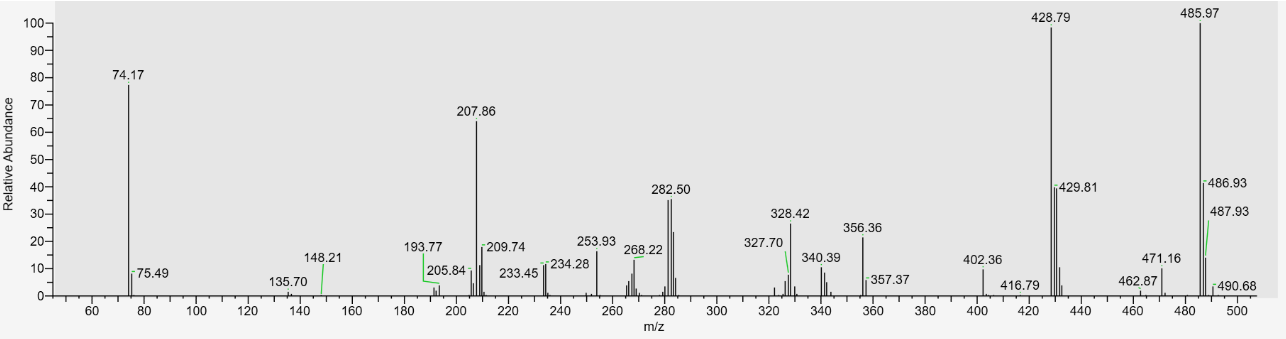

Original fragmentation spectra of compound 18 deriving from reaction A

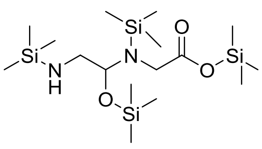

(**18**)

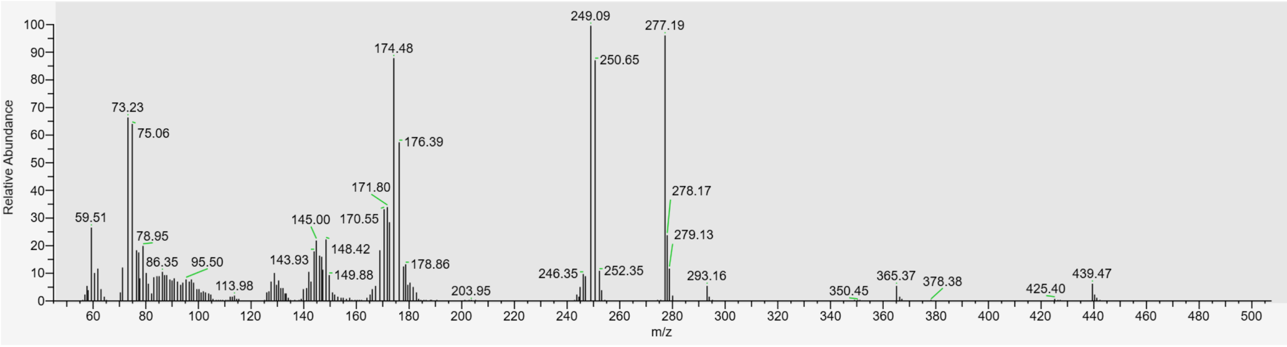

Original fragmentation spectra of compound 19 deriving from reaction B

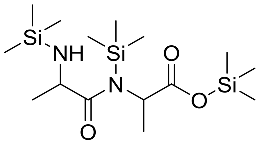

(**19**)

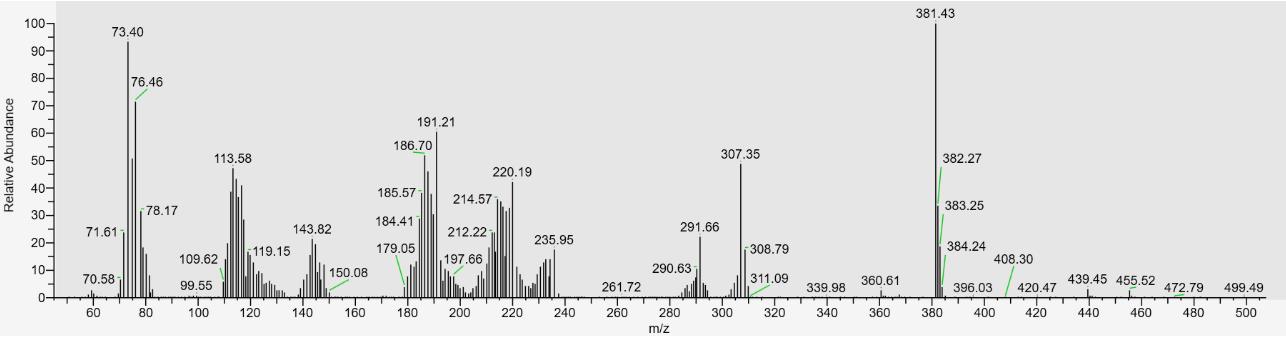

Original fragmentation spectra of compound 20 deriving from reaction C

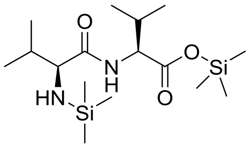

(**20**)

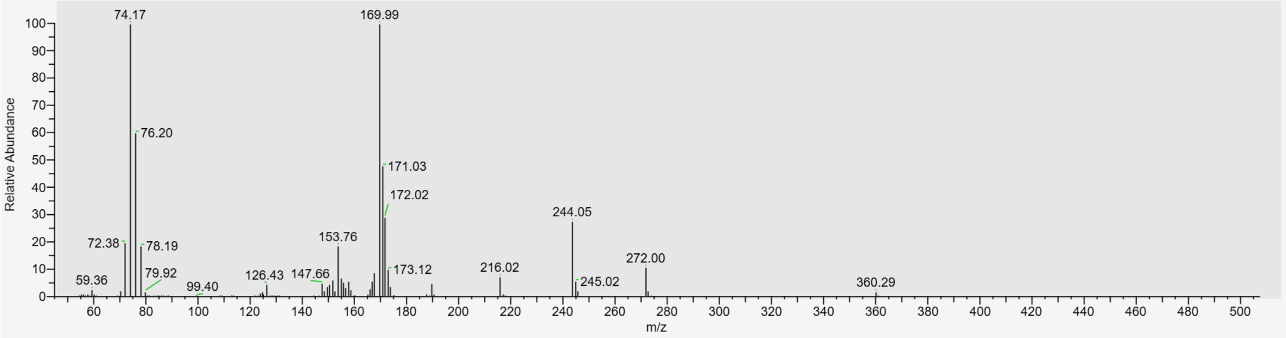

Original fragmentation spectra of compound 21 deriving from reaction D

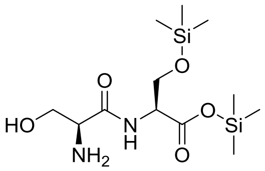

(**21**)

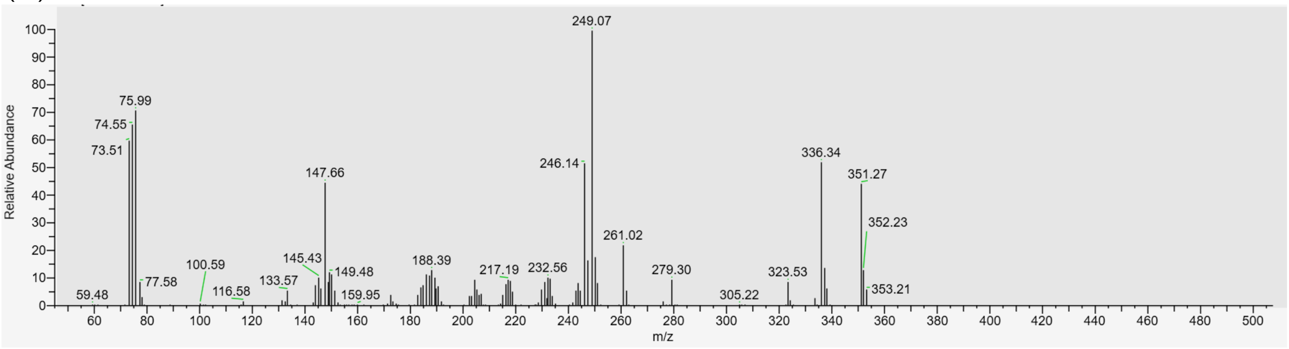

Original fragmentation spectra of compound 22 deriving from reaction A

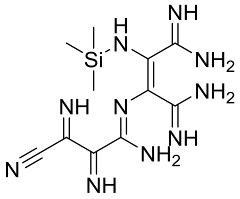

(**22**)

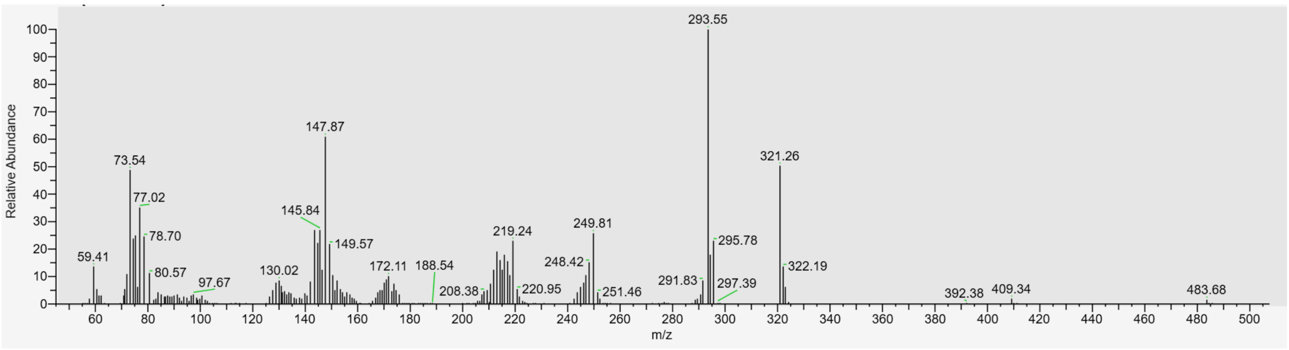

Original fragmentation spectra of compound 23 deriving from reaction A

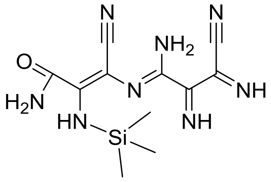

(**23**)

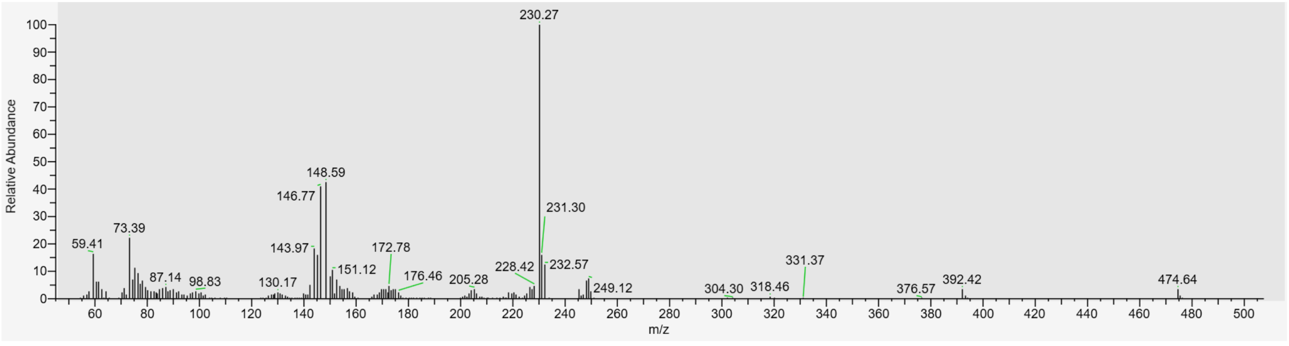

Original fragmentation spectra of compound 24 deriving from reaction A

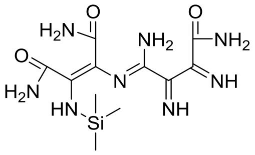

(**24**)

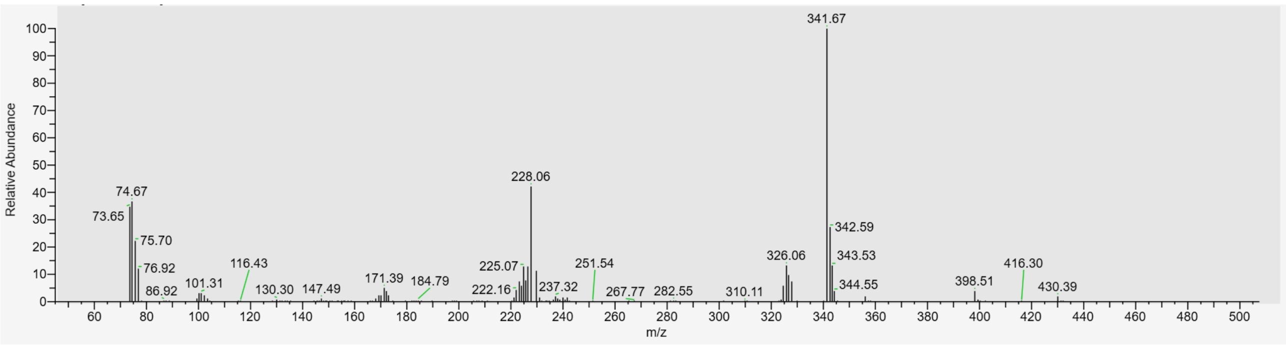

Original fragmentation spectra of compound 25 deriving from reaction A

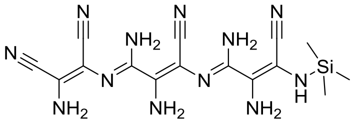

(**25**)

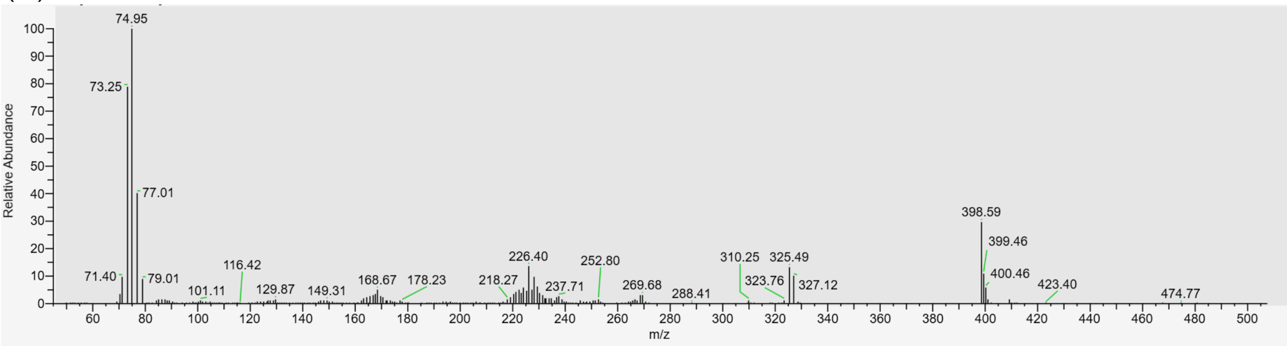

Original fragmentation spectra of compound 26 deriving from reaction A

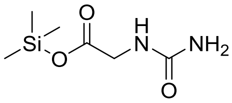

(**26**)

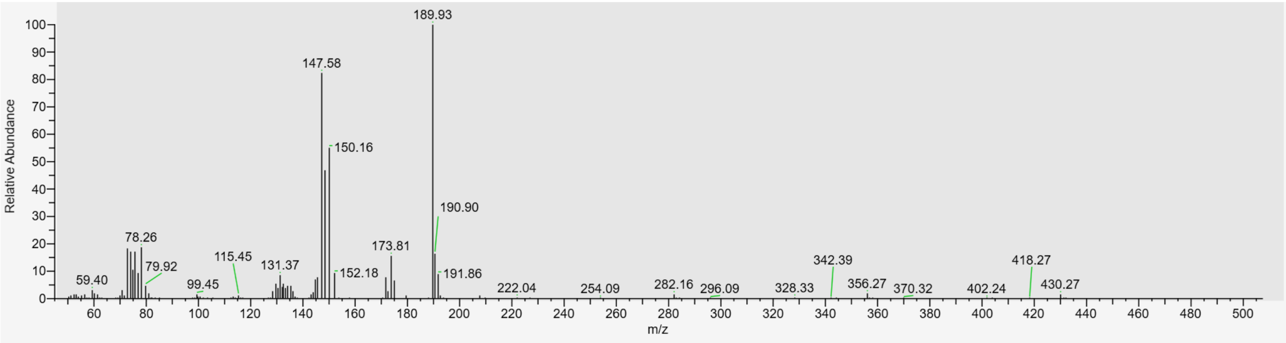

Original fragmentation spectra of compound 27 deriving from reaction A

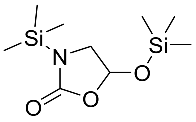

(**27**)

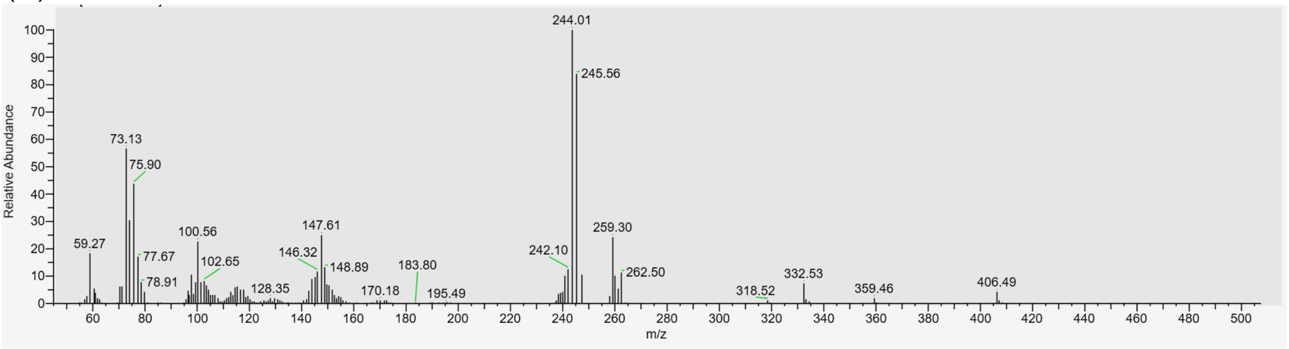

SI #4: m/z fragmentation spectra of standard compounds.

Original fragmentation spectra of standard compound 4

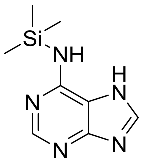

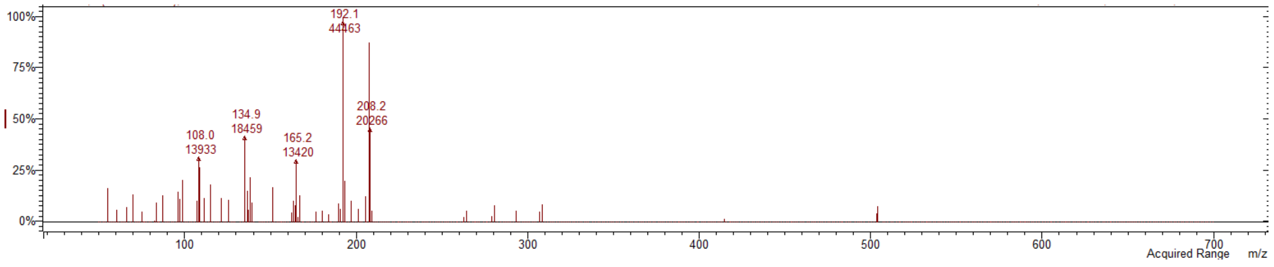

Original fragmentation spectra of standard compound 5

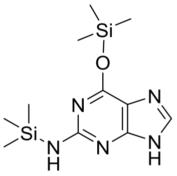

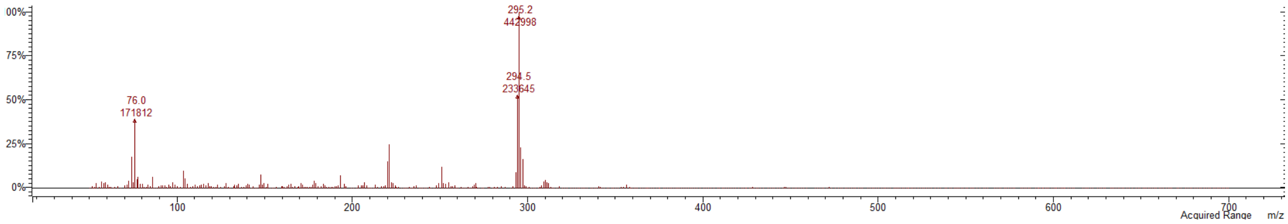

Original fragmentation spectra of standard compound 6

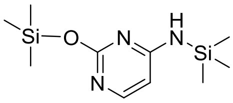

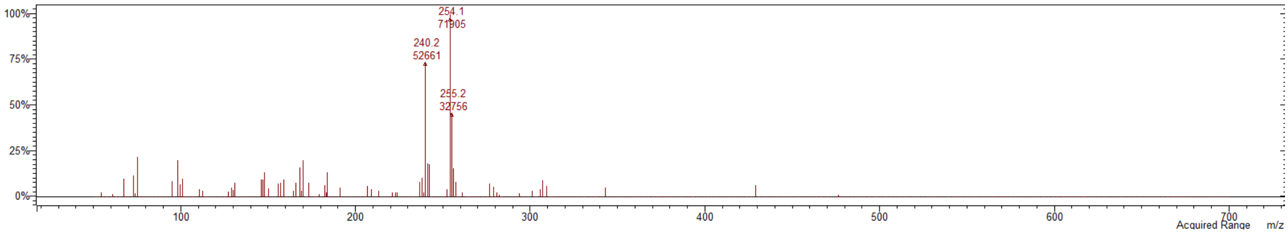

Original fragmentation spectra of standard compound 7

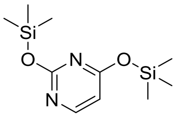

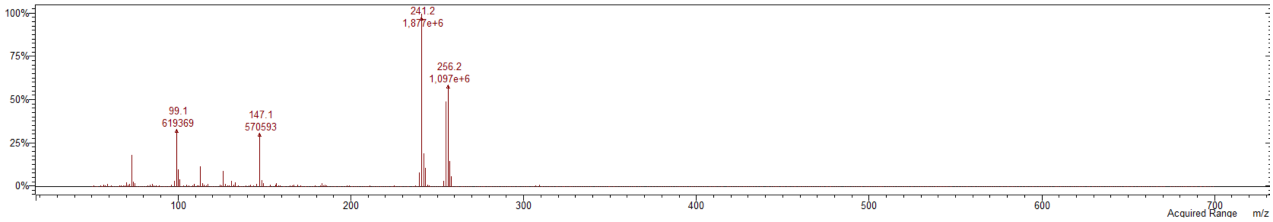

Original fragmentation spectra of standard compound 8

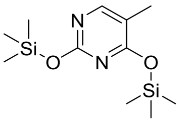

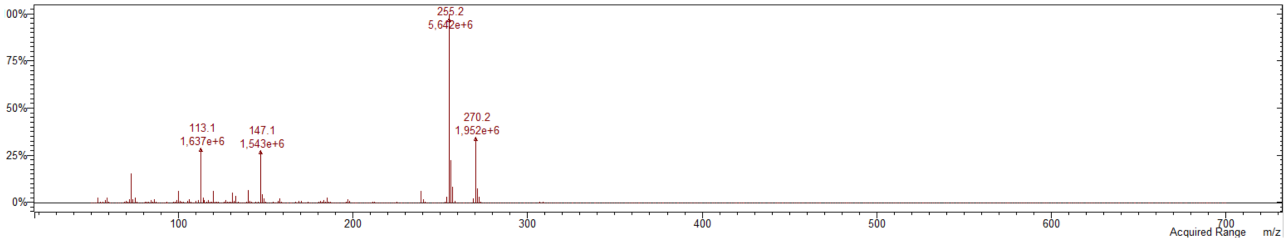

Original fragmentation spectra of standard compound 9

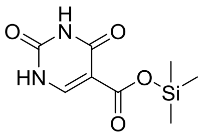

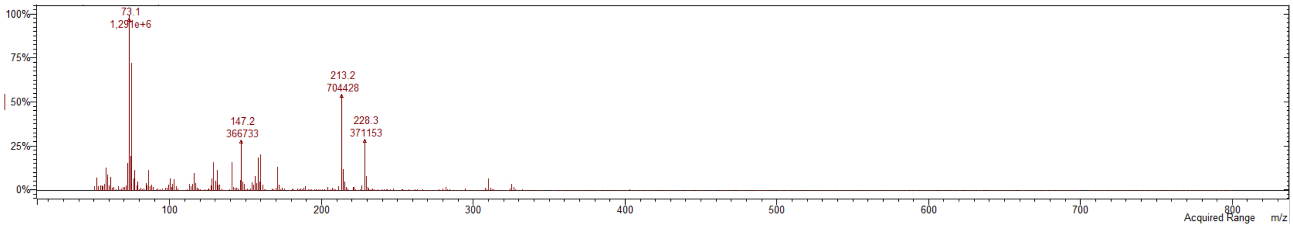

Original fragmentation spectra of standard compound 10

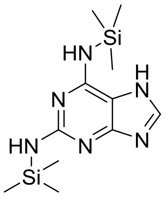

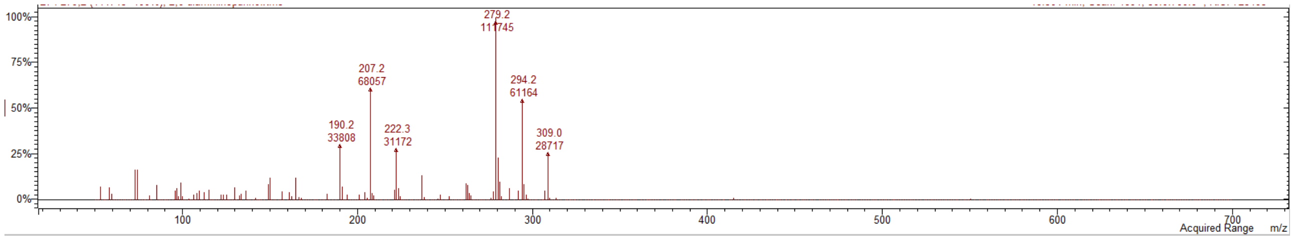

Original fragmentation spectra of standard compound 11

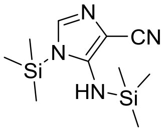

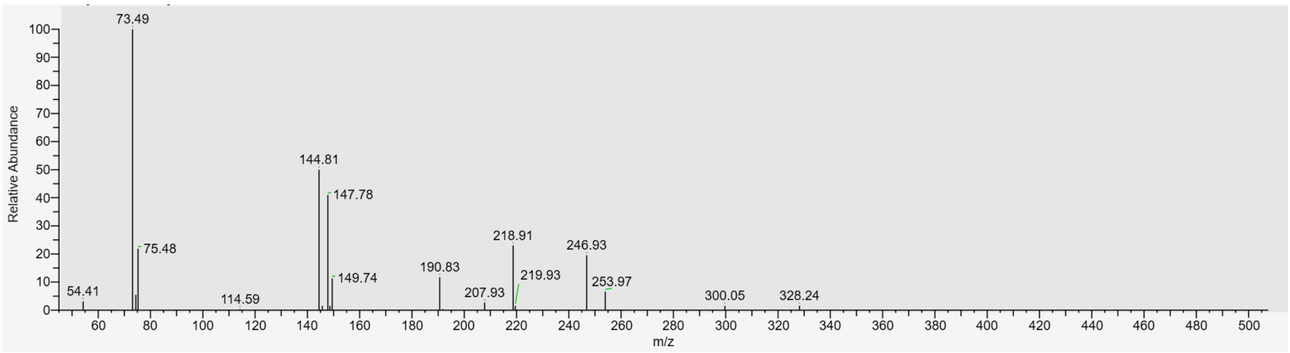

Original fragmentation spectra of standard compound 12

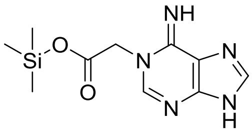

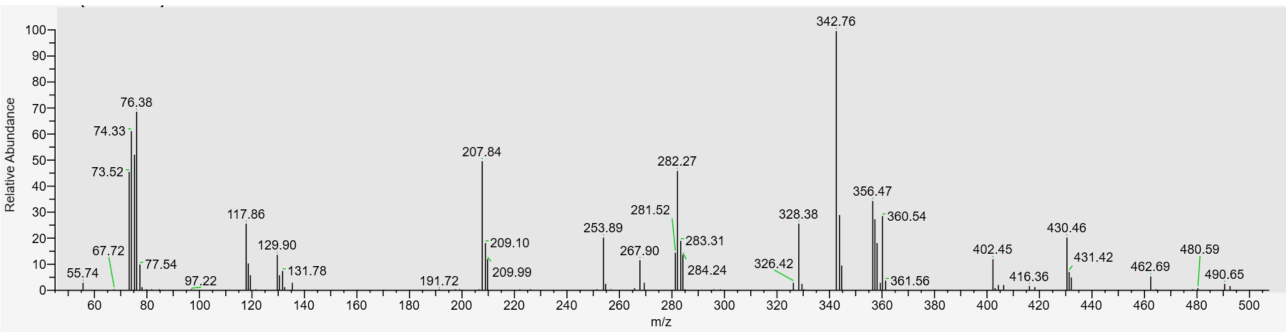

Original fragmentation spectra of standard compound 13

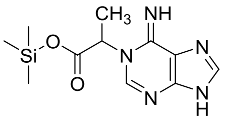

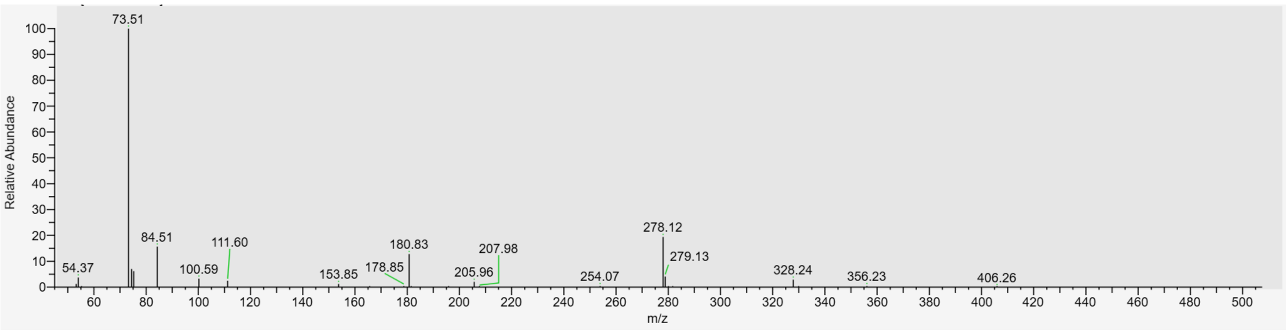

Original fragmentation spectra of standard compound 14

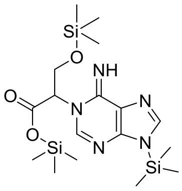

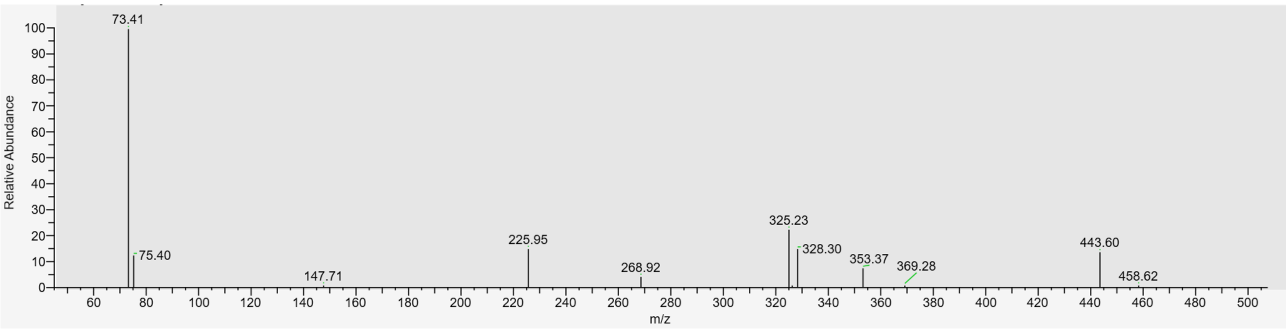

Original fragmentation spectra of standard compound 15

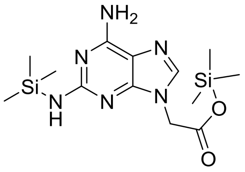

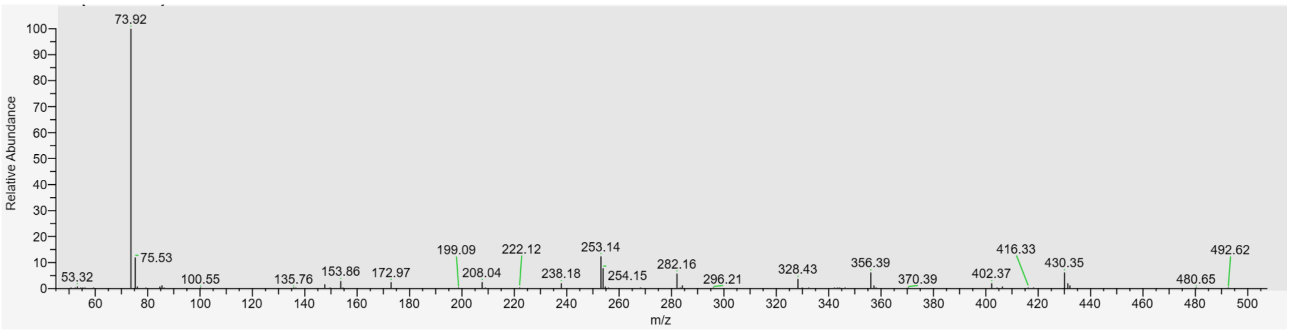

Original fragmentation spectra of standard compound 16

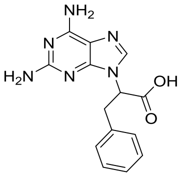

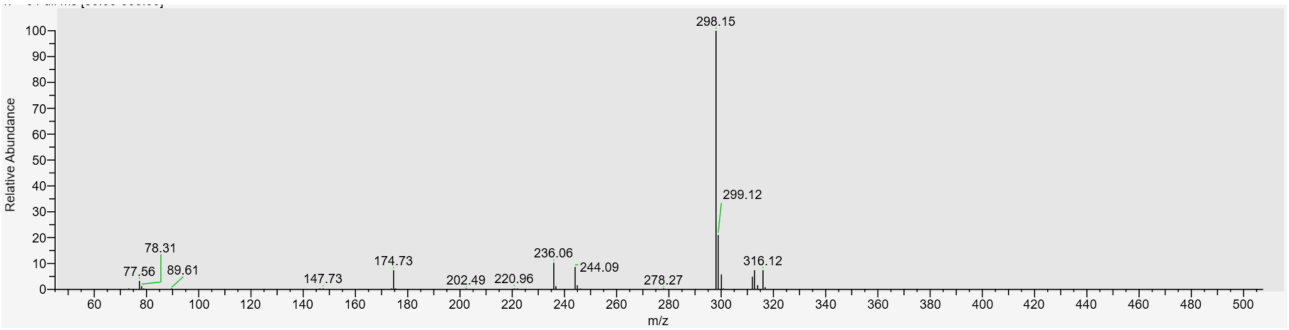

Original fragmentation spectra of standard compound 18

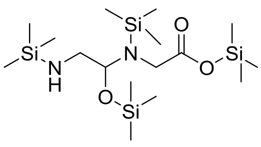

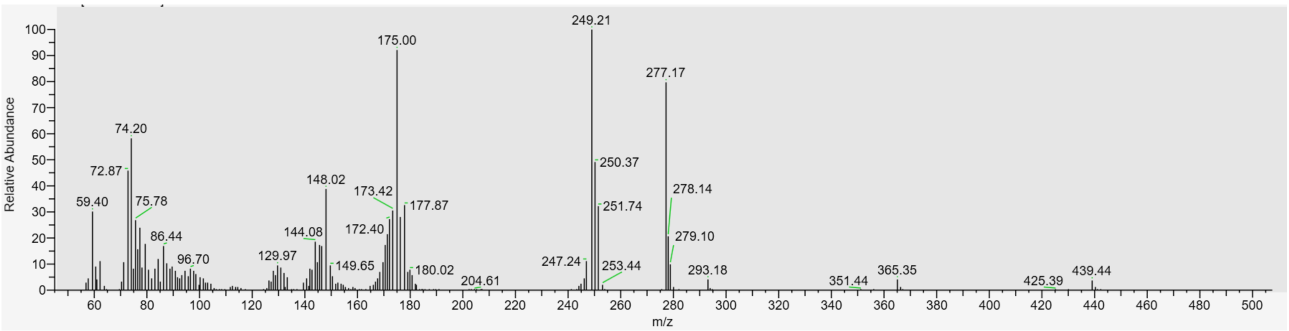

Original fragmentation spectra of standard compound 19

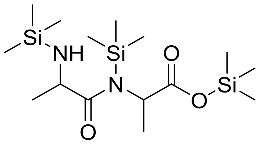

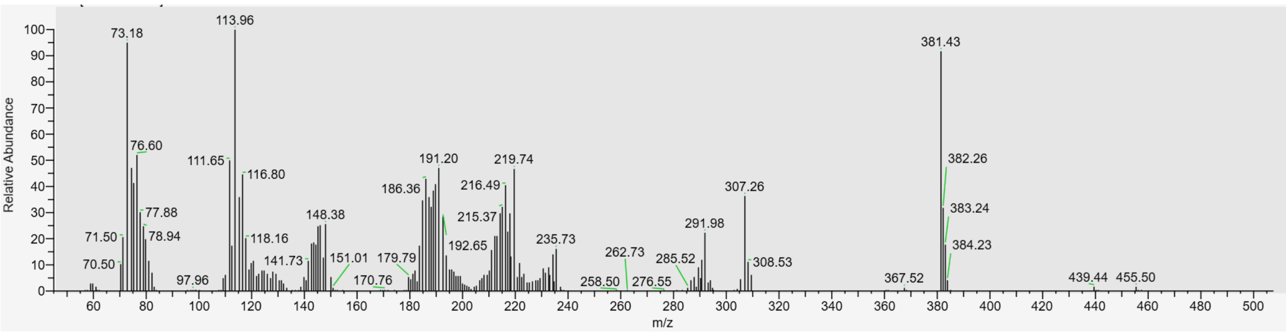

Original fragmentation spectra of standard compound 20

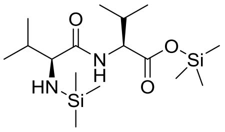

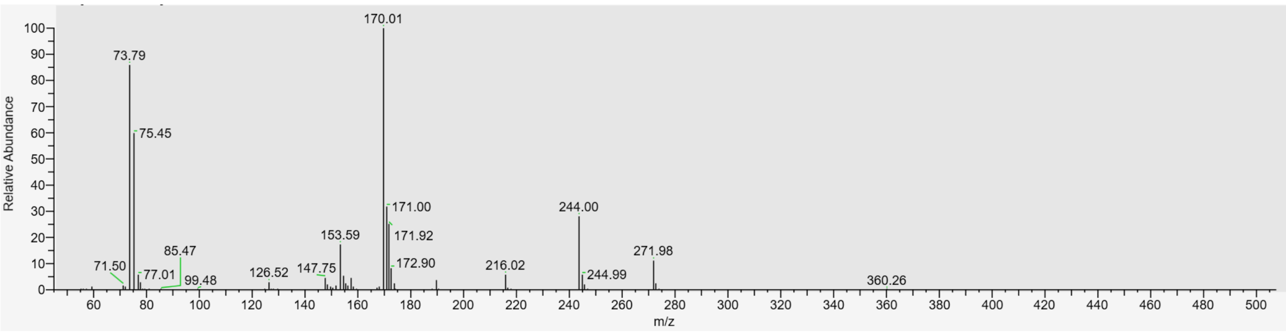

Original fragmentation spectra of standard compound 21

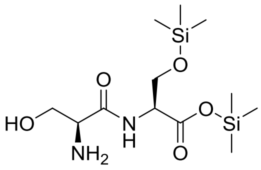

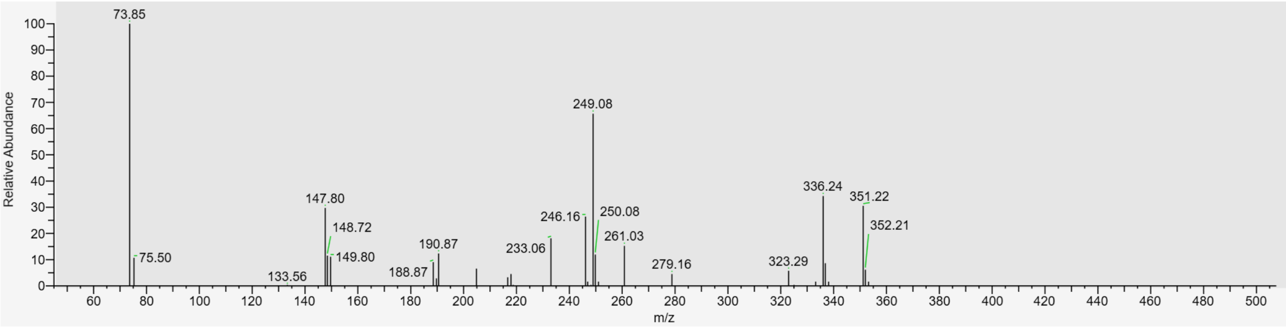

Original fragmentation spectra of standard compound 26

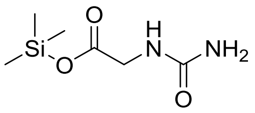

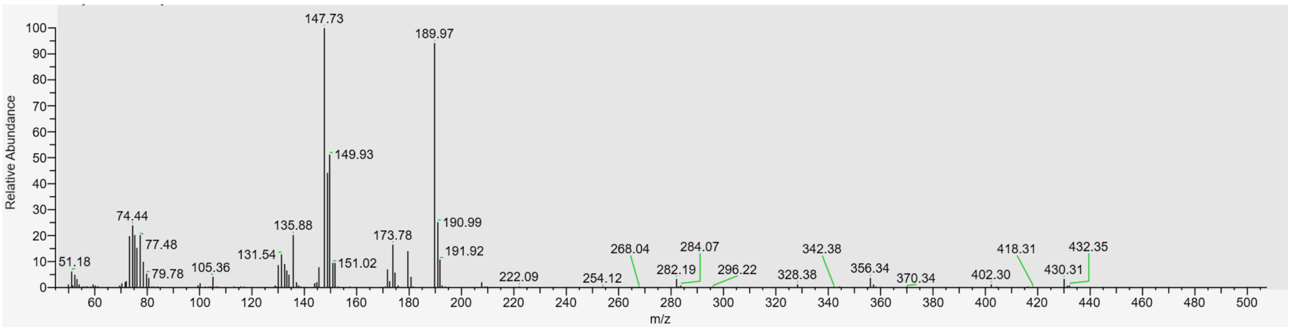

Original fragmentation spectra of standard compound 27

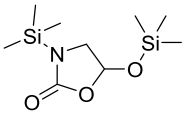

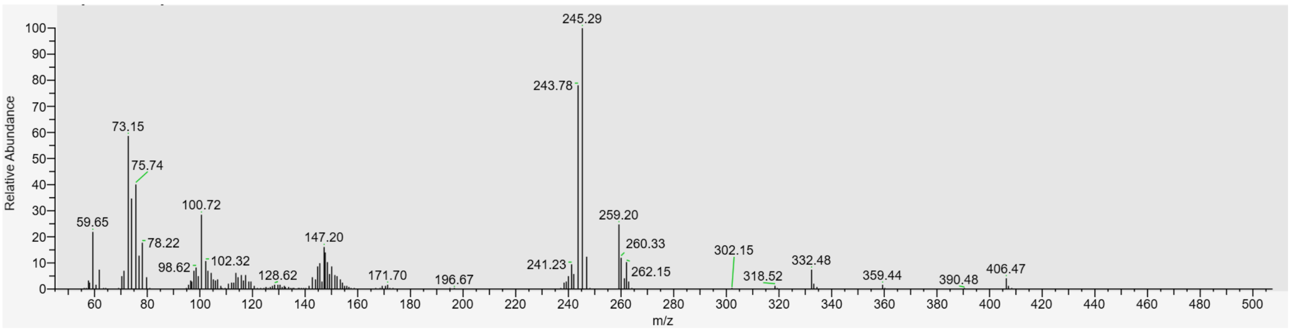

SI #5: Chromatograms of reaction A

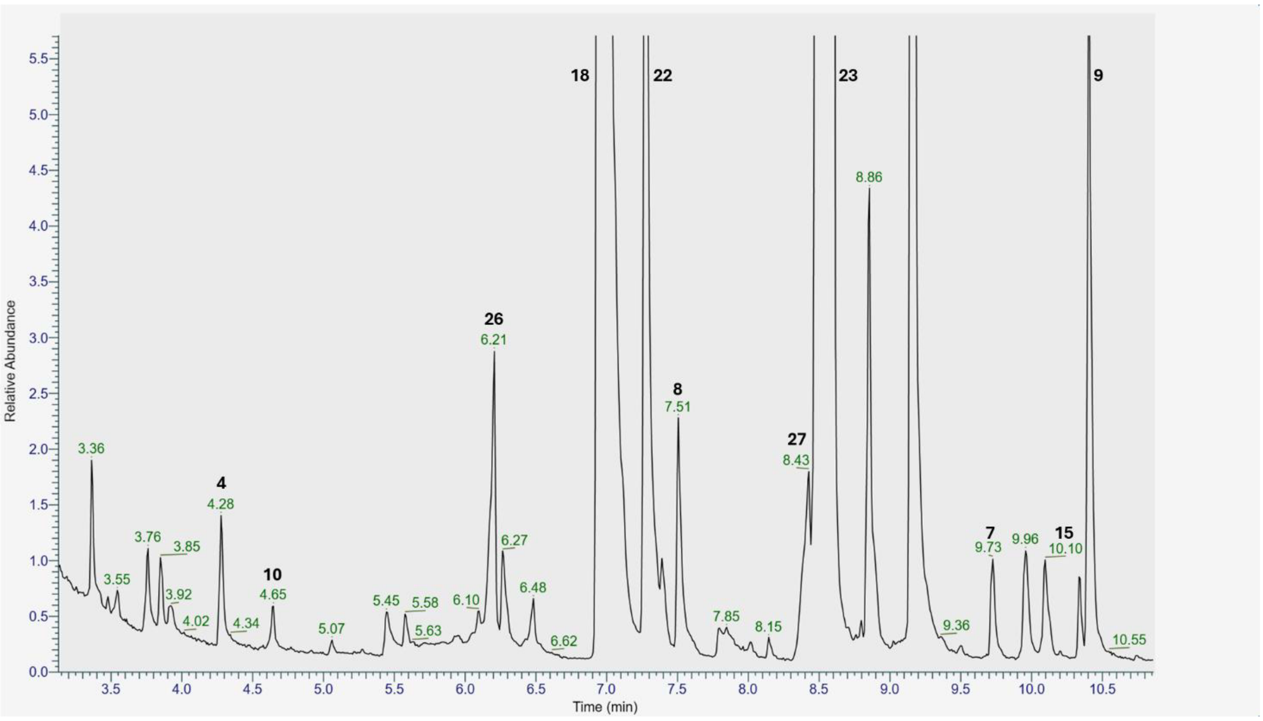

Figure S1, Panel A: 4: Adenine; 10: 2,6-diaminopurine; 26: Hydantoic acid; 18: Glycylglycine; 22: DAMN dimer; 8: Thymine; 27: N-carboxyamide (NCA); 23: DAMN dimer (1 hyd. Ac.); 7: Uracile; 15: N9-acetic acid-2,6-diaminopurine; 9: 5' COOH-uracile

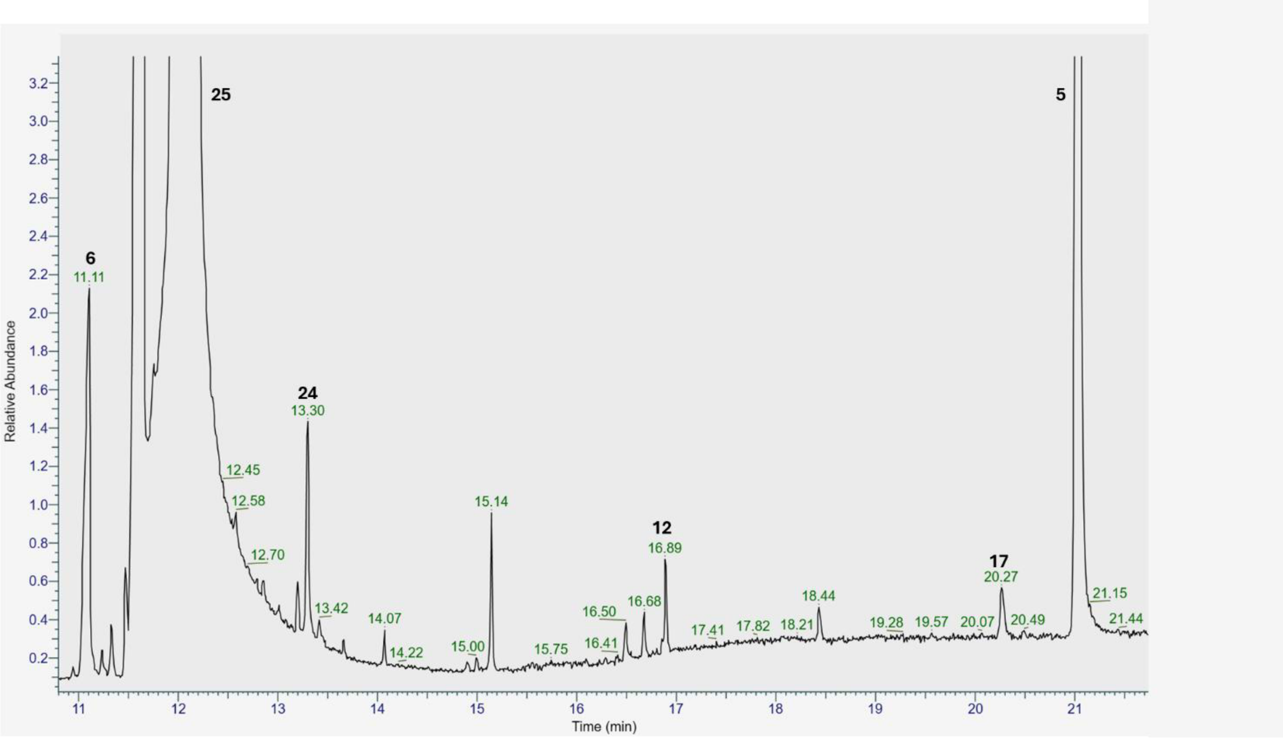

Figure S1, Panel B: 6: Cytosine; 25: DAMN trimer; 24: DAMN dimer (3 hyd. Ac.); 12: N1-acetic acid adenine; 17; 5: Guanine.

SI#6: in situ cGMP polymerization during the 2024 campaign

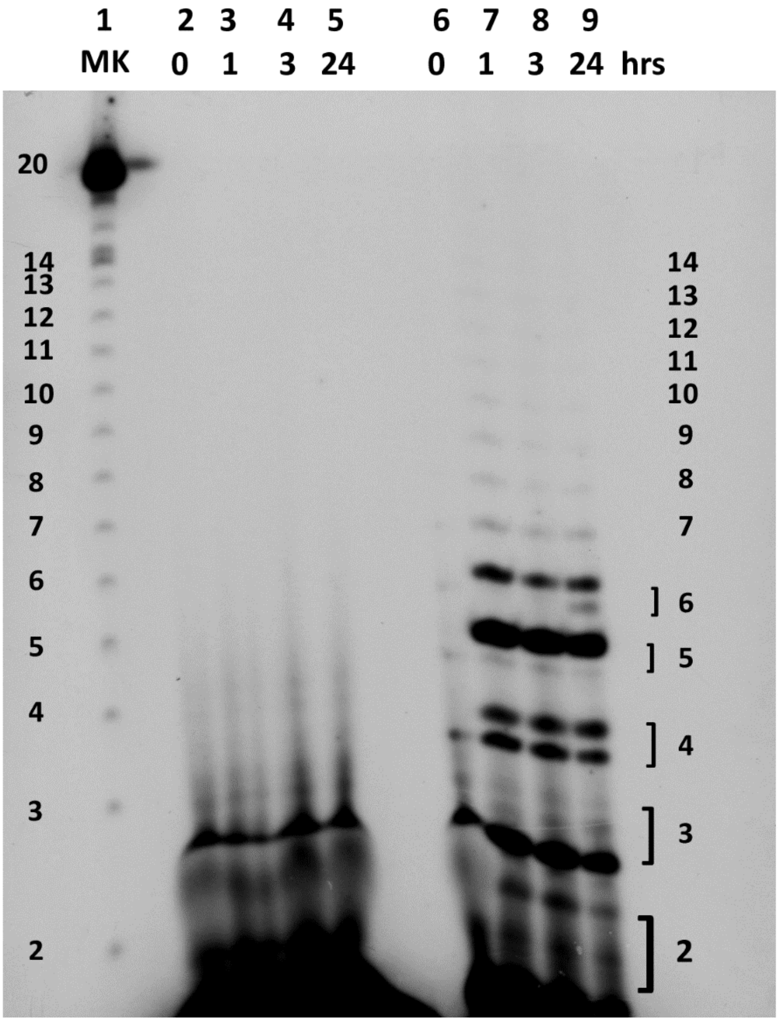

(a)

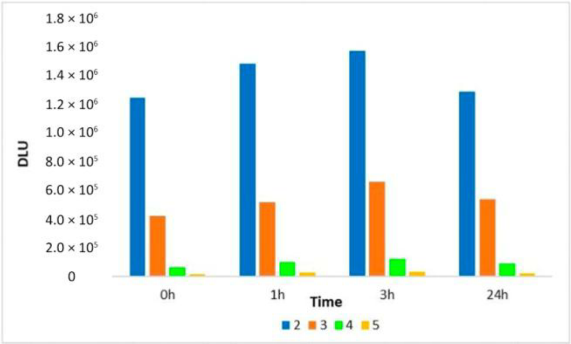

(b)

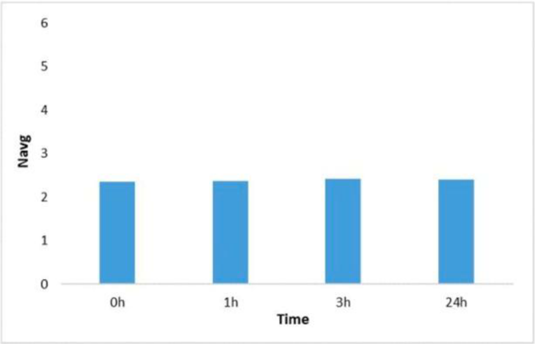

(d)

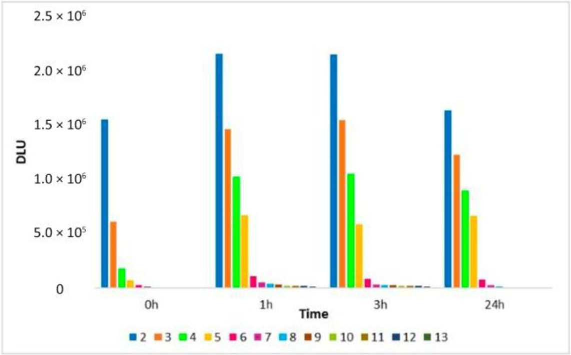

(c)

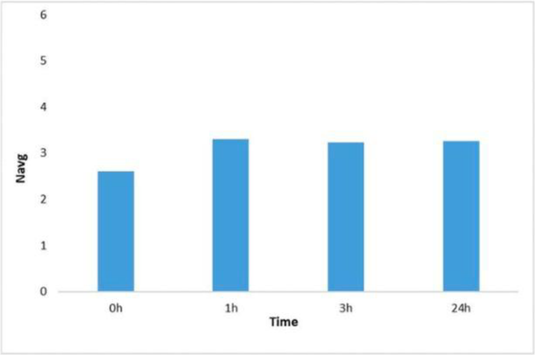

(e)

Figure S2. cGMP polymerization in situ in the Bagno dell'Acqua hot spring during the 2024 campaign. Lane 1: marker 5' ^{32}P-ribo G20. Lanes 2-5: samples resuspended in HPLC grade water (pH 5.5). Each sample was prepared by lyophilizing 150 μL of 1 mM 3',5' cGMP, resuspended in situ in 150 μL of HPLC grade water and subsequently incubated at 50°C in the thermal spring illustrated in Figure 1 HP for the indicated time. Each sample was treated and labeled independently. Lanes 6-9: samples resuspended in 150 μL of the lake water (filtered to 220 nm to remove contaminants, such as sediment and microbes). For the shortest oligomers two or three different hydrolysis products are observed (indicated by brackets). Panels (b) and (d): Variation of the signal intensity in digital light units (DLU) as a function of time for the samples showed in Panel (a). Length distribution of the polymerization products (length in nucleotide units, color coding is shown at the bottom of the panel) is indicated for each time point. Panels (c) and (e): the average length (Navg, in nucleotide units) of the polymerization products as a function of time for the samples showed in Panel (a). Constant production of dimers and trimers in decisively high amounts keeps this value low.

SI#7: Synthetic procedure and 1H.NMR data of compounds 12-16

General procedure for the synthesis of N-1 alkylpurine 12-14

To a solution of AICN (1.0 eq., 0.47 mmol) in CH_3CN (2 mL) was added ammonium formate (0.66 mmol, 1.4 eq.) and the appropriate amino acid as methyl ester (0.56 mmol, 1.2 eq). The reaction is left under stirring at 150°C for 24 hours. After the reaction returned to room temperature, the solvent was removed under reduced pressure. The crude was extracted using ethyl acetate (20 mL) and water (20mL). The organic layer was treated with Na_2SO_4 and concentrated under reduced pressure. The crude was treated with NaOH (1.0 N) and stirred for 18 h at room temperature. The solution was acidified with HCl 1.0 N until reaching neutral pH extracted with ethyl acetate and purified by flash chromatography. Compounds 12-14 were obtained in quantitative yield.

NMR Spectra of compound 12

^{1}H-NMR (400MHz, DMSO d_6, ppm): d 11.45 (s, 1H, NH), 8.44 (s, 1H, NH), 8.11 (s, 1H, CH), 7.08 (s, 1H, CH), 3.89 (d, J= 3 Hz, CH_2).

^{13}C-NMR (100 MHz, DMSO d_6, ppm): d 170.47 (C=O), 162.09 (C), 157.69 (C), 148.41 (C), 130.79 (C), 118.17 (C), 90.28 (C).

NMR Spectra of compound 13

^{1}H-NMR (400MHz, DMSO d_6, ppm): d 8.51 (s, 1H, CH), 8.02 (s, 1H, CH), 4.38-4.34 (m, 1H, CH), 1.26 (d, J= 3.6 Hz, CH_3).

^{13}C-NMR (100 MHz, DMSO d_6, ppm): d 173.13 (C=O), 164.94 (C), 157.69 (C), 142.1 (C), 116.0 (C), 49.97 (C), 46.62 (C), 17.55 (C).

NMR Spectra of compound 14

^{1}H-NMR (400MHz, DMSO d_6, ppm): d 9.76 (s, 1H, NH), 9.19 (s, 1H, NH), 8.27 (s, 1H, CH), 6.40 (s, 1H, CH), 6.20 (s, 1H, OH) 3.92 (d, J= 1.4 Hz, CH_2), 3.69 (s, 1H, CH).

^{13}C-NMR (100 MHz, DMSO d_6, ppm): d 174.18 (C=O), 151.8 (C), 150.3 (C), 147.6 (C), 144.8 (C), 118.0 (C), 67.3 (C), 42.5 (C).

General procedure for the synthesis of N-9 diaminopurines 15-16

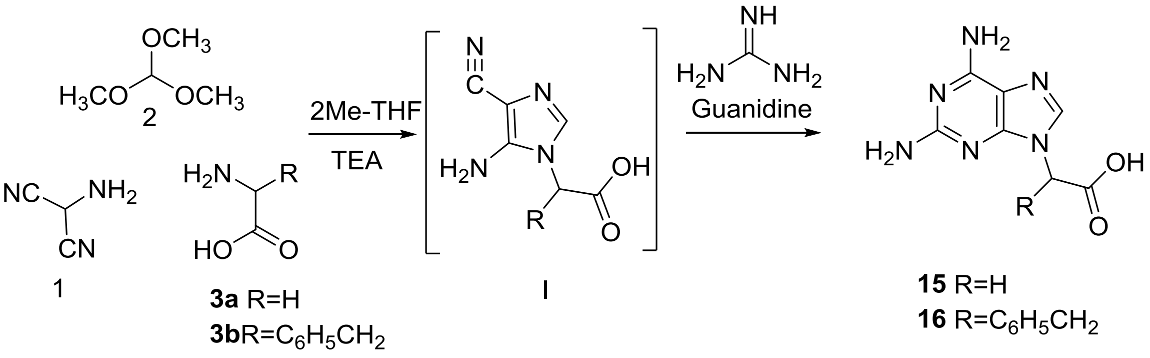

Aminomalononitrile p-toluenesulfonate 1 (5.9 mmol) dissolved in 2-MeTHF (30 mL) was treated with triethylamine (7.1 mmol) room temperature for 30 min, after which trimethyl orthoformate 2 (8.3mmol) was added, and the solution irradiated with microwave assistance using the program in Table below:

no. of cycles	temperature	Ramp time	Hold time	Pressure (psi)	Power (W)
1	200°C	1 min	2 min	250	250

Thereafter, the solution was cooled to room temperature, followed by the addition of triethylamine (7.1 mmol) and of the appropriate aminoacid 3a-b (glycine or phenylalanine methyl ester) (7.1 mmol). The solution was stirred under microwave conditions as described above. Thereafter, the solvent was removed, and the precipitate was dissolved in dichloromethane (30 mL) and extracted with saturated aqueous Na_2CO_3 (3 × 20 mL) and saturated aqueous NaCl (1 × 20 mL). The organic layer was treated with Na_2SO_4 and concentrated under reduced pressure. Purification was performed by flash chromatography with ethyl acetate (AcOEt)/hexane (Hex) (2:1) to afford imidazole intermediate I . Imidazole intermediate I (0.80 mmol, 1 equiv) and guanidine carbonate (1.60 mmol, 2 equiv) were irradiated under microwave conditions using the program in Table above. Thereafter, the solution was poured into hot water (8.0 mL), and the mixture was stirred for 10 min. After the mixture returned to room temperature, the solid residue was filtered, evaporated under reduced pressure, and purified by silica gel chromatography and eluting with 10% methanol in dichloromethane. The crude was treated NaOH (1.0 N, 1.0 mL) and stirred for 18 h at room temperature. The solution was acidified with HCl 1.0 N until reaching neutral pH, freeze-dried, and washed with methanol. The organic layer afforded 15 and 16 in quantitative yield after evaporation of the solvent.

NMR Spectra of compound 15

^{1}H-NMR (400 MHz, CD_3OD, ppm): δ 8.27 (s, 1H, CH), 4.45 (s,2H, CH_2).

^{13}C-NMR (100 MHz, CD_3OD, ppm): δ 169.03 (C=O), 160.05 (C), 155.87 (C), 152.92 (C), 147.19 (C), 115.74 (C), 49.74 (CH_2).

NMR Spectra of compound 16

^{1}H-NMR (400 MHz, CD_3OD, ppm): δ 8.28 (s, 1H, CH), 7.36−7.23 (m, 5H, CH-Ar), 5.21−5.16 (m, 1H, CH), 3.15−3.11 (dd, J =4.4, 14.0 Hz, 1H, CH_2), 3.03-2.97 (dd, J = 4.4, 14.0 Hz, 1H, CH_2).

^{13}C-NMR (100 MHz, CD_3OD, ppm): δ 169.04 (C=O), 156.95 (C), 154.78 (C), 152.15 (C), 149.98 (C), 141.92 (C), 129.58 (C-Ar x2), 127.82 (C-Ar x2), 124.88 (C-Ar), 112.64 (C), 64.45 (CH), 34.40 (CH_2).
